# Small Changes in Climate Can Profoundly Alter the Dynamics and Ecosystem Services of Tropical Crater Lakes

**DOI:** 10.1371/journal.pone.0086561

**Published:** 2014-01-31

**Authors:** Émilie Saulnier-Talbot, Irene Gregory-Eaves, Kyle G. Simpson, Jackson Efitre, Tobias E. Nowlan, Zofia E. Taranu, Lauren J. Chapman

**Affiliations:** 1 Department of Biology, McGill University, Montréal, Québec, Canada; 2 Department of Biological Sciences, School of Biosciences, College of Natural Sciences, Makerere University, Kampala, Uganda; University of Waikato (National Institute of Water and Atmospheric Research), New Zealand

## Abstract

African tropical lakes provide vital ecosystem services including food and water to some of the fastest growing human populations, yet they are among the most understudied ecosystems in the world. The consequences of climate change and other stressors on the tropical lakes of Africa have been informed by long-term analyses, but these studies have largely focused on the massive Great Rift Valley lakes. Our objective was to evaluate how recent climate change has altered the functioning and services of smaller tropical lakes, which are far more abundant on the landscape. Based on a paired analysis of 20 years of high-resolution water column data and a paleolimnological record from a small crater lake in western Uganda, we present evidence that even a modest warming of the air (∼0.9°C increase over 20 years) and changes in the timing and intensity of rainfall can have significant consequences on the dynamics of this common tropical lake type. For example, we observed a significant nonlinear increase (*R^2^_adj_* = 0.23, *e.d.f.* = 7, *p<*0.0001) in thermal stability over the past 20 years. This resulted in the expansion of anoxic waters and consequent deterioration of fish habitat and appears to have abated primary production; processes that may impair ecosystem services for a vulnerable human population. This study on a system representative of small tropical crater lakes highlights the far-reaching effects of global climatic change on tropical waters. Increased research efforts into tropical aquatic ecosystem health and the development of sound management practices are necessary in order to strengthen adaptive capabilities in tropical regions.

## Introduction

The paucity of reliable long-term environmental and climatic data sets for the African continent is hindering progress in building capacity to adapt to global change [1], particularly in aquatic systems where potential impacts of climate change are not adequately documented and understood [2], [3]. Given the range of uncertainty surrounding climate change scenarios and increasing pressures facing African inland waters [4] including a continent-wide shortage of potable water [5], the development of local scale data sets is critical for these systems [6]. Here, we address this problem by presenting an analysis of a 20-year high-resolution record of regular monitoring in a small but locally significant equatorial crater lake, Lake Nkuruba, located in a densely populated rural region of the East African highlands.

**Figure 1:**
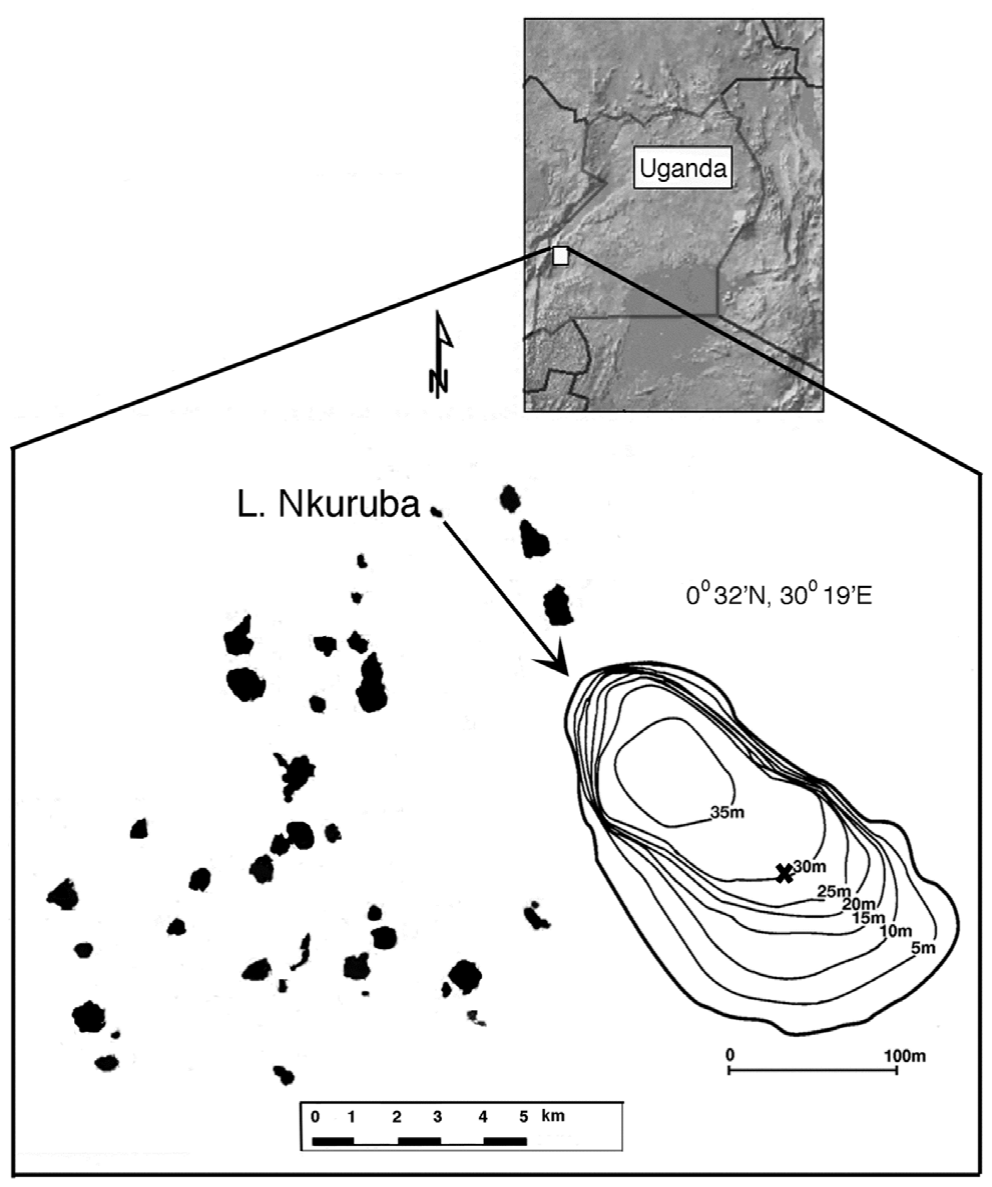
Map showing location of the study lake among other crater lakes of the Kabarole, Uganda region. The X marks the coring site and contour lines represent bathymetry (modified from [15]).

Crater lakes are a common lake type in the tropics [7]. About 90 crater lakes of varying sizes dot the landscape of western Uganda, one of the most densely populated rural areas in Sub-Saharan Africa [8]. Many of these lakes are relied upon for subsistence fishing and water supply for livestock and domestic use. Some, like Lake Nkuruba (Fig. 1), also provide additional sources of income for local populations (in this case, support for an orphanage in the form of a small-scale communal ecotourism venture; http://www.enfuzicommunitycampsite.com/). In this region, crater lakes have often experienced water quality degradation driven by watershed land-use changes [9]. However, our study lake’s catchment, which is limited to the surrounding crater walls up to the rim, has been the focus of an integrated conservation development program implemented in the early 1990s that resulted in the quick return and maintenance of the forest on the slopes to a near pre-disturbance state. As such, the basin’s forest has remained largely intact over the past 20 years. Consequently, cultural eutrophication threats are presently low in Lake Nkuruba, making it an ideal system for studying effects of recent climate change on small, warm tropical lakes, without the confounding effects of eutrophication.

Long-term studies of the much larger Lake Tanganyika show that 20^th^ century climate warming, which is unprecedented in 1500 years [10], has modified water column dynamics, decreased ecosystem productivity and altered biotic assemblages [11], [12]. Tanganyika is the largest African lake by volume and directly supports the livelihoods of millions of people [11]. However, small lakes are also of critical importance to humans throughout the tropics [13], and yet have received comparatively little attention from researchers. The present study provided a rare opportunity to both explore and assess impacts of recent climate warming on a smaller tropical lake ecosystem with high-resolution data that commence in 1992.

## Results and Discussion

**Figure 2:**
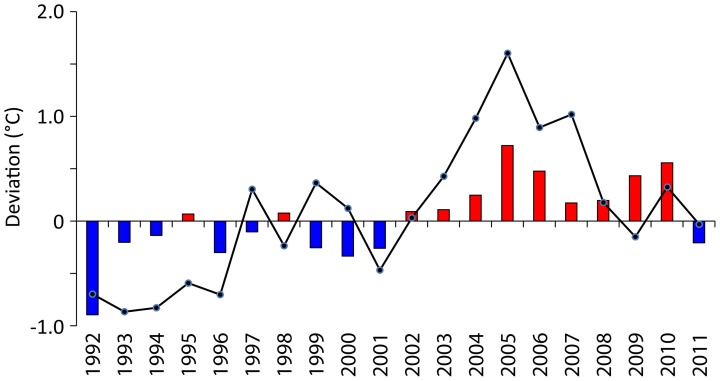
Air (line) and surface water temperature (bars) anomalies for Lake Nkuruba over 20 years. Anomaly computed as the difference from the 1992–2011 mean air and surface water temperatures.

**Figure 3:**
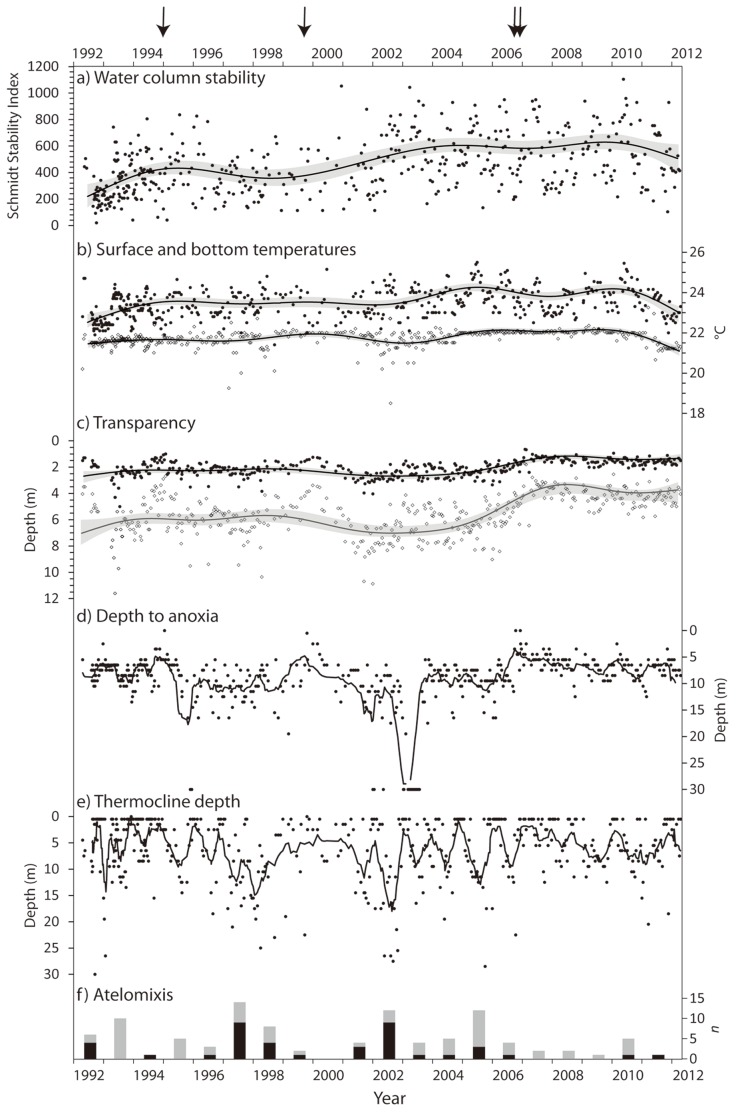
Water column data in Lake Nkuruba 1992–2012. Arrows indicate the timing of observed mixing events. a) thermal stability of the water column at the time of sampling expressed using the Schmidt Stability Index computed following [27]; line indicates trend; b) surface (black circles) and bottom (open diamonds) water temperatures; c) Secchi (black circles) and photic zone (open diamonds) depths (photic zone = Secchi×2.7 [28]); d) depth to anoxia determined as the depth at which dissolved oxygen measurements drop below 1 mg L^−1^; e) thermocline depth determined as the depth where the greatest inflection in the temperature curve occurs over a thickness of 1 m; f) annual occurrences of atelomixis events identified by thermocline depths greater than 15 m (black bar) or situated between 15 and 10 m (grey bar). For a–c) lines and grey bands indicate the nonlinear (GAM) response curves and the 95% confidence intervals, respectively. For d–e) lines indicate a 10-point running mean.

On a global scale, mid- and high-latitude northern hemisphere lakes are exhibiting much more pronounced warming than tropical lakes [14]. However, because the rate of change in water density is greater at higher temperatures [13], warm equatorial lakes are more sensitive than temperate lakes to small increases in temperature. Since the early 21^st^ century, Lake Nkuruba has been subject to warmer than average temperatures. Indeed, nonlinear modeling indicates that between 2002 and 2010 surface water temperatures consistently showed a positive anomaly from the 1992–2012 mean (*R^2^_adj_* = 0.22, *e.d.f.* = 8.6, *p*<0.0001; Fig. 2). Likewise, we detected increased stratification of the water column over time, indicated by higher than average values of the Schmidt Stability Index post 2006 (SSI; Fig. 3a; *n* = 478, *R^2^_adj_* = 0.23, *e.d.f.* = 7, *p*<0.001). The strong positive correlation of thermal stability and surface water temperature in Nkuruba (*n* = 26, *r* = 0.93, *p*<0.001 [15]; and *n* = 478, *r* = 0.55, *p* = 0.0001, this study) explains in part this increase. The relationship between SSI and surface temperature was found to be non-linear (sigmoidal: *R^2^_adj_* = 0.29, *e.d.f.* = 3.7, *p*<0.0001). Bottom temperatures also increased significantly (about 0.5°C) and became more stable between 2002 and 2010 (*R^2^_adj_* = 0.24, *e.d.f.* = 8.7, *p*<0.0001; Fig. 3b), consistent with late 20^th^ century observations in Lake Malawi [16]. Ensuing these changes in thermal structure, with a lag of several years, we detected a transition to less transparent waters (Secchi and photic depth: *R^2^_adj_* = 0.33, *e.d.f.* = 8.1, *p*<0.0001; Fig. 3c) and shallower depth to anoxia post 2006 (defined as *z*_DO_ <1 mg L^−1^) (*R^2^_adj_* = 0.32, *e.d.f.* = 8.6, *p*<0.0001; Fig. 3d). Since late 2006, Secchi (transparency) readings have consistently been below the 20-year mean value (2 m), signifying a reduction in the depth of the underwater photic zone (Fig. 2c). Annual thermocline depth and depth to anoxia were correlated to Secchi depth (*n* = 20_(annual means)_, *r* = 0.51, *p* = 0.02 and *r* = 0.68, *p*<0.001, respectively) and to each other (*n* = 20_(annual means)_, *r* = 0.69, *p*<0.001). These variables show a trend of shallower and more stable values for this period (Fig. 3).

Because hydrographic features, particularly water column structure and circulation, affect nutrient cycling and can dominate annual patterns of phytoplankton seasonality in the tropical belt [17], changes to the vertical structure of the water column triggered by sustained warmer temperatures will almost certainly have an impact on the lake’s biota. Over the entire monitoring period, we observed only four episodes of thermal and/or oxic homogenization, defined as sampling days with either <0.2°C amplitude in the water column and/or <1 mg L^−1^ DO at the lake’s surface (Table 1). These observations provide clear evidence of oligomixis, which had previously been presumed [17], [18], but rarely recorded in this lake type. More commonly detected in Lake Nkuruba were large increases in the thickness of the mixed layer, indicated by variability in the depths of the thermocline (Fig. 3e). In Nkuruba, deeper thermoclines are significantly more likely to occur during the dry seasons (i.e. between early December through to late February and between approximately late May through to early September) than during the wet seasons (i.e. early March through late May and September to November; *t* = 1.98, *p* = 0.05), despite muted seasonality in these equatorial waters. Termed “atelomixis”, this partial mixing is thought to be very important in increasing the productivity of tropical lakes by bringing up nutrients from deep water to offset nutrient depletion during stratification periods [19]. Coincident with the changes in the Schmidt stability data, we observed a large decrease in the number of days showing evidence of atelomixis (i.e. ∼13% of sampling days between 2006–2012, compared to an average of 27% observed prior to 2006). The observed reduction in the frequency and in the intensity of atelomixis since 2006 (Fig. 3f) could limit this source of enrichment and thus lead to decreased efficiency in nutrient use [13] and decreased primary production.

**Table 1:** Observed mixing events in Lake Nkuruba 1992–2012.

Date of observation	Surface temperature	Δ Temp surface-bottom	Surface DO (mg L^−1^)	Δ DO surface-bottom (mg L^−1^)
1994-12-03	21.9	0.15	0.4	0.3
1999-08-30	22	0	1.5	1.3
2006-08-10	23	1.0	0.6	0.5
2006-10-05	23.2	1.55	1.0	0.9

Mixing events are defined as sampling days with either <0.2°C amplitude in water column and/or ≤1 mg L^−1^ DO at surface. All temperatures are in °C.

**Figure 4:**
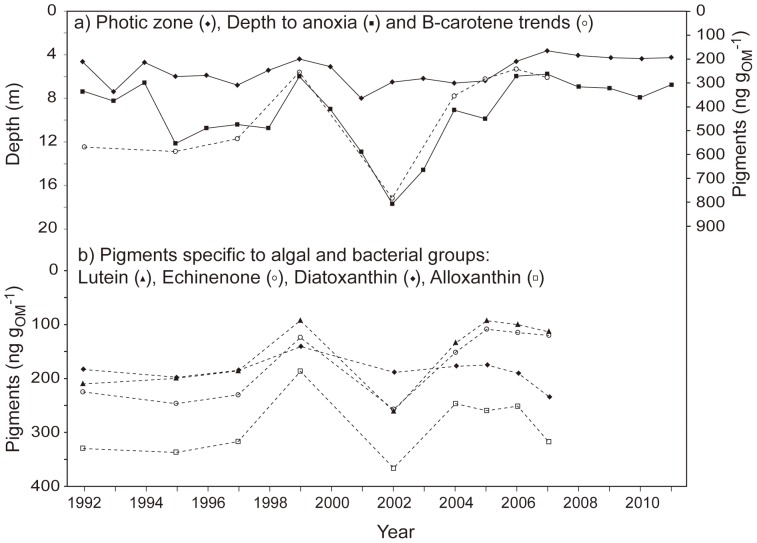
Water column dynamics in Lake Nkuruba 1992–2010. a) annual means of depth to anoxia (squares) and transparency (diamonds), and inferred primary production (circles) expressed as sedimentary concentrations of β-carotene. b) 1992–2007 variations in the concentrations of four sedimentary pigments associated with diatoms (diamonds), cryptophytes (triangles), chlorophytes (squares *concentration values for sedimentary lutein were an order of magnitude higher than the others, and were divided by 10 to fit the graph) and cyanobacteria (circles).

In the absence of regularly monitored observations of autochthonous production in Lake Nkuruba, we adopted the paleolimnological approach to explore the response of the lake biota to changes in water column structure. We compared the trends in our 20-year data set with the sedimentary pigment record from a core taken in 2008. We found a significant correlation between sedimentary β-carotene values (a relatively stable pigment reflecting total algal biomass [20]) and annual variation in depth to anoxia (*n* = 9, *r* = 0.84, *p* = 0.02) (Fig. 4a). The coherency of low values of sedimentary β-carotene and a shallower depth to anoxia strongly suggest that less mixing of the water column decreases the recycling of nutrients in the photic zone and negatively affects primary production. Data for other pigments representing the major algal and bacterial groups present in the lake (Fig. 4b) generally follow a similar trend, showing an overall coherent response to the physical changes in the lake over the sampling period. Therefore, within the timeframe of the 20 year monitoring period, the few data points available for the period subsequent to 2006 highlight the importance of tracking primary productivity to evaluate whether it continues to decrease in the context of prolonged warmer conditions.

**Figure 5:**
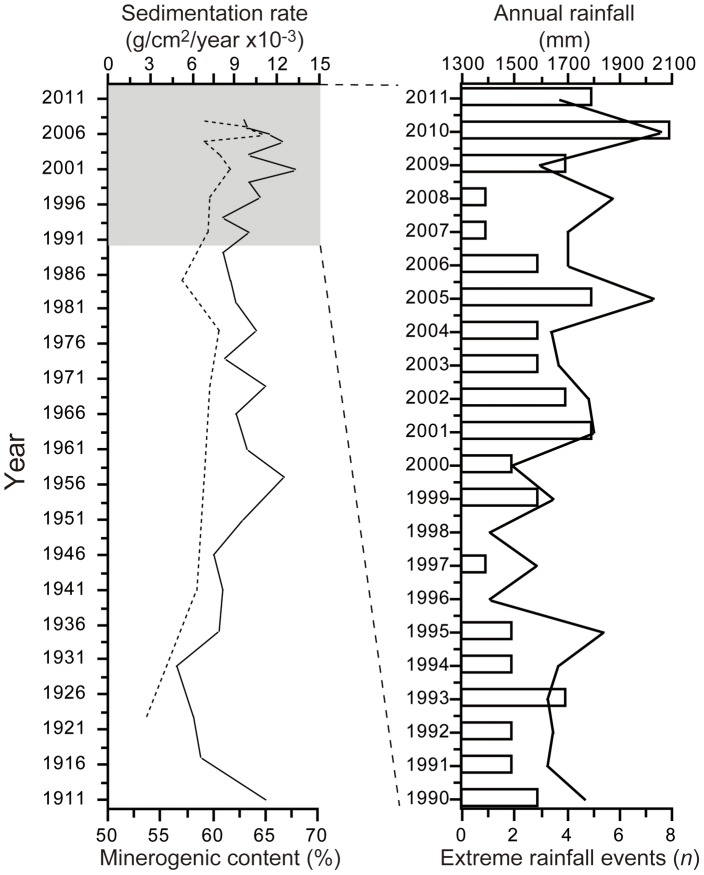
Sediment accumulation rate, minerogenic content, recent extreme rainfall events. a) Sediment accumulation rate (dashed line) in the Nkuruba core (in gr/cm^−2^/year; rates are comparable to other crater lakes from the region, Saulnier-Talbot unpublished) and minerogenic content (the unburnt fraction of the sediment after LOI) of the sediment; number of extreme rainfall events per year and total annual precipitation over the sampling period (data modified from [8]).

The decline in water transparency that accompanies the observed decrease in primary production to date (Fig. 4a) is perhaps counter-intuitive. One might expect that reduced productivity in the lake would equate to higher transparency, as it does in Lake Tanganyika [12]. However, the Nkuruba region has experienced a recent increase in days with heavy rainfall (i.e. days receiving ≥50 mm of rain, as measured in nearby Kibale National Park; *R^2^_adj_* = 0.41, *e.d.f.* = 5.1, *p* = 0.05). As such, greater sediment loads that negatively affect water transparency is a viable hypothesis to explain the decline in water transparency in this small lake without any surface inflow other than rainfall. Enhanced sediment loading is supported by higher sedimentation rates detected in the core in the mid-2000s, relative to the previous 80 years (Fig. 5). The significant relationship between years with extreme rainfall events and proportion of minerogenic input in the core (*n* = 10, *r* = 0.68, *p* = 0.03) is also concordant with this hypothesis (Fig. 5). A shift in the vertical distribution of the lake’s planktonic assemblage could also explain the recent decline in transparency. Studies of Nkuruba’s phytoplankton indicate that there is an important peak in the abundance of the cyanobacteria that dominate the assemblage (>70·10^6^L^−1^ cells), situated in the lower epilimnion at the limit of the oxic zone [21]. A shallower epilimnion is likely causing an upward migration of this peak, thus potentially contributing to reduced water transparency. This mechanism may be common in tropical crater lakes as suggested by the strong correlation between mixing depth and water column transparency observed previously in the tropical crater lakes of Cameroon [18]. The effects of the recent changes in the water column structure of our study lake on the specific composition of its phytoplankton assemblage are still unknown; however, from the sedimentary pigment data (Fig. 4b), it does not appear that a recent change in the composition of planktonic assemblages has occurred (at the group level).

**Figure 6:**
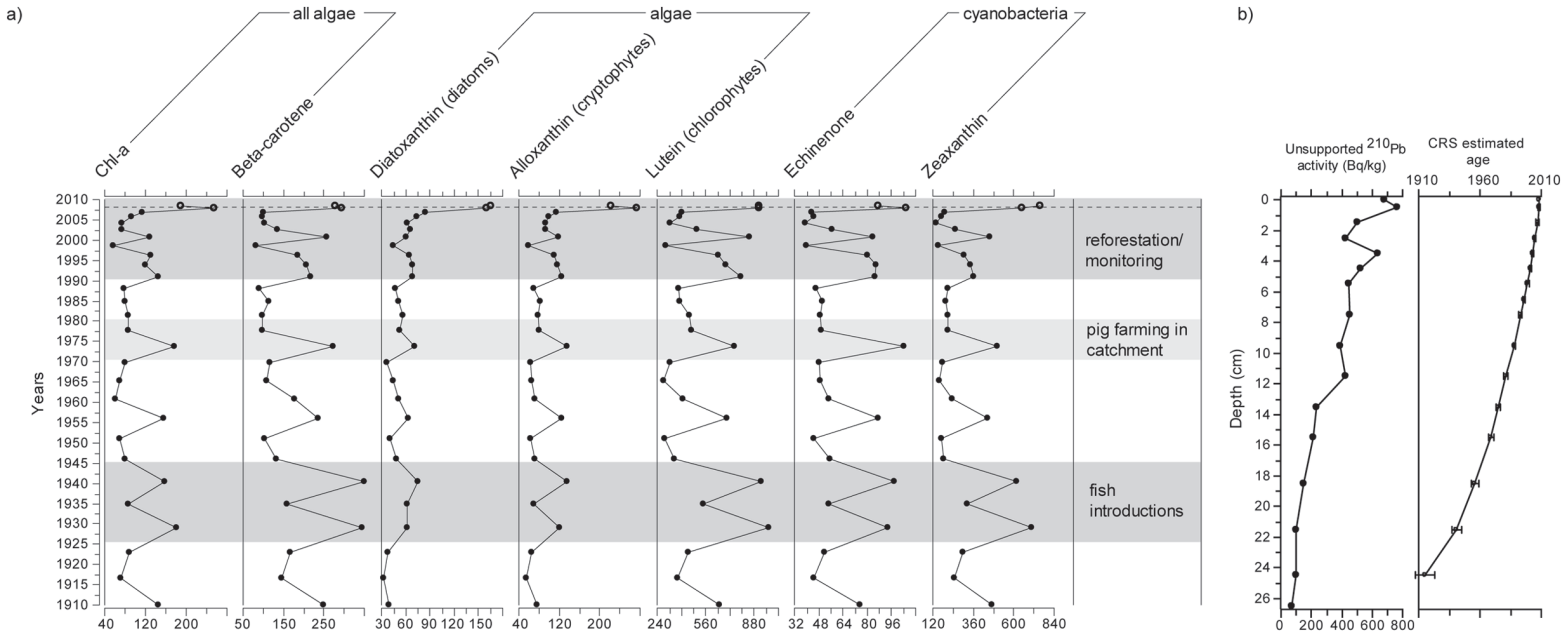
Sedimentary pigment variations (1910–2008) and core chronology. a) Historical variations in selected pigments and important 20^th^ century human interventions in the Lake Nkuruba catchment. The dotted horizontal line indicates that the uppermost data points (shown as empty circles) were not considered in our analyses due to differences in digenesis between these and older samples [29]. b) Unsupported ^210^Pb concentrations in 16 samples and constant rate of supply (CRS) model used to determine downcore ages (including error bars).

The longer temporal context provided by the entire sediment core shows that productivity in the lake has undergone major fluctuations over the past 100 years (Fig. 6a). Given the substantial history of anthropogenic disturbances in the lake’s catchment pre-1990, it is not possible to directly attribute earlier variations in sedimentary pigments to climatic mechanisms. Many of the Ugandan crater lakes, including Lake Nkuruba, host populations of non-native tilapias introduced into the lakes in the 1940s and later to boost protein availability for the local communities [21], [22]. The timing of the fish introductions seems to correspond to the most pronounced increase in primary productivity over the last century (however, this did not lead to a major increase in the sedimentation rate at the time). This suggests that changes in algal abundance in the last 20 years could in part be due to changes in fish community structure. Several of the lakes are currently producing “stunted” tilapia populations [9], [22]. Some also suffer periodic fish kills due at least in part, to severe short-lived hypoxia events triggered by mixing of the anoxic hypolimnion with the surface water or the expansion of the anoxic zone, such as the one recently observed in our study lake. Furthermore, in Lake Nkuruba, herbivores are mostly consumed by invertebrate predators that are favoured by anoxia [21], which could ultimately lead to lowered fish yield per unit of primary production. Environmental predictors of fish condition in the Ugandan crater lakes are not straightforward [22], but a prolonged period of warmer than average temperatures will affect fish habitat availability and quality. Detrimental conditions for tilapias in Nkuruba, and possibly in other nearby lakes would further reduce reliability of the regional protein supply for local human populations and would require managers to rethink the viability of some of the ecosystem services that have been provided until recently by the Ugandan crater lakes. In lakes such as Nkuruba, with long water residence times and where internal recycling is a major process, human use and management of the lake and its catchment can have a lasting impact on present functioning, which may only recently have been affected by climate change.

## Conclusions

Our long-term observations show that small changes in climate, in the form of sustained warmer-than-average annual temperatures and more heavy rainfall days, are a major driver of change in Lake Nkuruba. We provide evidence that small, deep tropical crater lakes can show effects arising from climate warming that are comparable to those described for the African Great Lakes, namely abated production due to reduced mixing, hinting towards widespread concern for the general health of aquatic ecosystems in East Africa. We still know very little about the resilience of tropical lakes to global change, and it is difficult to predict if Lake Nkuruba has crossed over into a kind of alternative steady state, or if the present conditions could easily be reversed with the onset of a sustained cooler period. For now, it appears that one cooler than average year (2011) did not alter the depth to anoxia or thermocline depth. Clearly, African crater lakes should be better monitored so that hypotheses about the future impacts of climate change can be tested. In tropical regions of Africa, such as western Uganda and Cameroon where crater lakes are prevalent and provide local populations with ecosystem services, problems of food, water and energy security due to rapid population increases could be exacerbated by the effects of climate change on the dynamics of these ecosystems.

## Materials and Methods

Nkuruba (Fig. 1) is a small (3 ha), moderately deep (max *z* = 38 m, mean *z* = 16 m), slightly alkaline (pH = 8), freshwater (mean surface salinity = 360 µS cm^−1^) maar lake. It is surrounded by high forested walls averaging 48 m above the water surface which shelter the lake from wind action. It has no apparent inflow or outflow, and water level changes are relatively small but were observed to fluctuate considerably (falling over 3 m) following an earthquake in February 1994 [15]. Permission and all necessary permits were obtained for the described study, which complied with all relevant regulations, from the appropriate Uganda government authorities (Uganda National Council for Science and Technology).

We measured water temperature, dissolved oxygen, and transparency in the water column of Lake Nkuruba roughly bi-monthly between March 1992 and February 2012, with a period of reduced sampling frequency (monthly) between August 1998 and June 2001, for a total of 489 sampling days. In general, duplicate profiles of dissolved oxygen and water temperature were taken with a YSI Model 50 or 51B meter, calibrated on each sampling day. Values presented are averages of the two profiles measured at each depth. Transparency was measured with a 22 cm Secchi disk by two different people, and then averaged. Measurements were generally taken before local noon at a station in the middle of the lake, where maximum depth reaches 30 m to 38 m. Sampling efforts were occasionally hindered by the presence of wildlife (hippopotamus) and the location of the profile was moved slightly, though it remained in the deeper zone. Previous sampling protocols of 6 sites across the lake during a continuous period of 24 months (between July 1992 and July 1994) indicated significant but very low variation in dissolved oxygen across sites (<0.5 mg L^−1^). Therefore, we used only the one central lake profile throughout the 20 year period. Air temperatures and rainfall were measured at the Makerere University Biological Field Station (MUBFS), located in nearby Kibale National Park.

A 28 cm-long sediment core was retrieved from the deepest part of the lake using a gravity corer in January 2008. The core was extracted vertically on site into 1-cm increments using a portable extruder. At all times during sediment processing care was taken not to expose the sediments to direct sunlight. Samples were kept in the dark and frozen until analyses were performed at McGill University’s Department of Biology.

Sedimentary pigments were analyzed in September 2008. A known pre-weighed amount of roughly 0.3 g (±0.0127 g) of freeze-dried sediment per subsample was treated. Pigments were isolated/extracted into 5 ml (final volume) of 100% acetone, and pre-processed by sonication in an ice bath for heat dissipation. Sample vials were purged of oxygen/air and placed in an atmosphere of argon. The extraction was allowed to continue for 24 h at −20°C. Sediments were separated from the supernatant by centrifugation in a refrigerated unit and filtration through 2 µm PTFE filters. Aliquots were analyzed using high performance liquid chromatography [23] (Waters HPLC, model no 600/626 with a Waters Photodiode Array 2996, a Waters 2475 Multi λ Fluorescence detector and a refrigerated waters autosampler 717). Individual pigments were identified and quantified using external standards purchased from DHI (Denmark). Sedimentary pigment concentrations were calibrated relative to organic matter content. Organic matter and minerogenic content was measured by loss on ignition [24].

Sediment core chronology was based on the measurement of ^210^Pb, ^214^Pb, and ^214^Bi from a known mass and volume of freeze-dried sediment from 16 downcore intervals. Using these measurements, we applied the constant rate of supply model (CRS) to estimate sediment ages [25] (Fig. 6b). All radiometric measurements were made using a Canberra well-detector gamma spectrometer over a 24 hr period. Given that there was approximately a one-year error associated with sediment ages for the past 20 years, the correlation between monitoring and paleolimnological time series were improved when we accounted for this error.

We considered linear and non-linear relationships between variables of interest. In particular we ran generalized additive models (GAMs) to test for the significance of a smoother term using the *mgcv* package for R [26]. Akaike Information Criterion (AIC) with a Chi-square test on model deviance was used to evaluate whether linear or nonlinear relationships had stronger statistical support. The effective degrees of freedom (e.d.f.; also known as the effective number of parameters of the cubic spline smoother) presented here indicate the amount of smoothing, such that an e.d.f. value very close to 1 suggests that the relationship is linear. We also ran an Analysis of Variance and post-hoc Tukey test to determine whether there was a significant difference in thermocline depth between the wet and dry seasons.
